# Application of multivariate chemometrics tools for spectrophotometric determination of naphazoline HCl, pheniramine maleate and three official impurities in their eye drops

**DOI:** 10.1038/s41598-023-46940-0

**Published:** 2023-11-11

**Authors:** Khadiga M. Kelani, Maha A. Hegazy, Amal M. Hassan, Mahmoud A. Tantawy

**Affiliations:** 1 Pharmaceutical Analytical Chemistry Department, Faculty of Pharmacy, Cairo University, Kasr el Aini Street, Cairo, 11562 Egypt; 2 Analytical Chemistry Department, Faculty of Pharmacy, Modern University for Technology and Information, El-Hadaba El-Wosta, Mokatam, 5th District, Cairo, Egypt; 3 Chemistry Department, Faculty of Pharmacy, October 6 University, 6 October City, Giza, Egypt

**Keywords:** Analytical chemistry, Spectrophotometry

## Abstract

This work is concerned with exploiting the power of chemometrics in the assay and purity determination of naphazoline HCl (NZ) and pheniramine maleate (PN) in their combined eye drops. Partial least squares (PLS) and artificial neural network (ANN) were the chosen models for that purpose where three selected official impurities, namely; NZ impurity B and PN impurities A and B, were successfully determined. The quantitative determinations of studied components were assessed by percentage recoveries, standard errors of prediction as well as root mean square errors of prediction. The developed models were constructed in the ranges of 5.0–13.0 μg mL^−1^ for NZ, 10.0–60.0 μg mL^−1^ for PN, 1.0–5.0 μg mL^−1^ for NZ impurity B and 2.0–14.0 μg mL^−1^ for two PN impurities. The proposed models could determine NZ and PN with respective detection limits of 0.447 and 1.750 μg mL^−1^ for PLS, and 0.494 and 2.093 μg mL^−1^ for ANN. The two established models were compared favorably with official methods where no significant difference observed.

## Introduction

Determining drug purity for ensuring its safety and quality is considered essential step in drug analysis. As a result, impurities detection and quantification take a great attention in pharmaceutical industries^1^. The demand to establish analytical methods which have the ability to determine and quantify the drug along with its impurities was consequently evoked^2^. On the other hand, as the number of the analyzed drugs and their impurities increased, the difficulty of their analysis using conventional methods was also amplified, taken in our consideration the simplicity and the availability of the spectrophotometric methods than the chromatographic ones^3–8^. In such case, multivariate data inspection is better in finding an answer to that complicated matrices^9, 10^. Therefore, chemometrics is commonly used to analyze such complex data obtained from spectrophotometric measurements to acquire valuable information. It is a useful tool when determining numerous drugs in their combined pharmaceutical formulations is required^11, 12^.

Naphazoline HCl (NZ), also known as 2-(naphthalen-1-ylmethyl)-4, 5-dihydro-1H-imidazole; HCl, is a drug that acts through decreasing pulmonary congestion. It reacts with α-adrenergic receptors located in the conjunctiva, producing a sympathomimetic effect leading to decrease swelling and edema of the eyes^13^. Reviewing its pharmacopeial monographs^14, 15^, shows that its quantification is achieved using two liquid chromatographic methods. Four reported impurities, namely; A, B, C and D, are also stated in its British pharmacopoeia (BP) monograph. Carefully reviewed spectrophotometric techniques reveal that NZ has been quantified in the existence of other substances^16–21^.

Pheniramine maleate (PN) is an alkylamine antihistaminic drug having anti-cholinergic properties through binding to H1 histaminic receptors. It is a first-generation drug which inhibits phospholipase-A2 and cyclic-GMP levels^22^. Its assay in the United States pharmacopoeia (USP) and BP is through high performance liquid chromatographic (HPLC) technique. In addition, two impurities, A and B, are reported in its BP monograph^14, 15^. A spectrophotometric method based on complexation with ferric ion has been published for its simultaneous determination with colorpheniramine maleate^23^.

**Figure 1:**
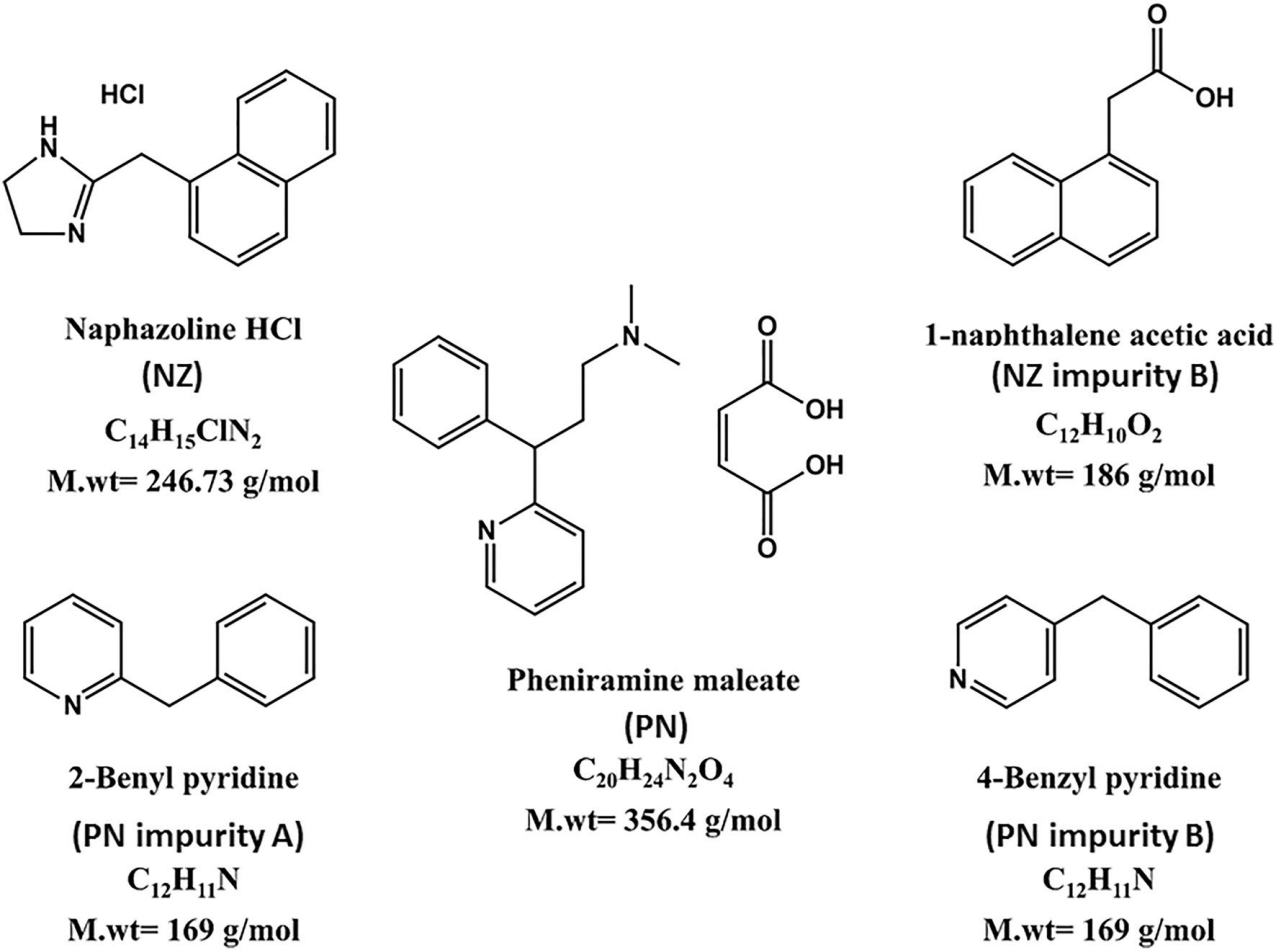
Chemical structures of the five cited components.

For the treatment of inflammatory eye disorders and allergic conjunctivitis, the approach of using NZ and PN together in combined eye drops is found to be more useful than using each drug alone^24^. Literature review reveals that NZ and PN have been quantified together using HPLC^25–28^, capillary electrophoresis^29^ and thin layer chromatography^30^. Two of these chromatographic methods have been published by our research group where their determination along with three reported official impurities, NZ impurity B, PN impurity A and PN impurity B, was achieved^28, 30^. In spite of the applicability of the chromatographic technique in analyzing such complex mixtures, it is still considered challenging. This is attributed to the requirements for the successive sample pretreatment steps as well as the selection of suitable mobile phase and stationary phase to attain the best separation and system suitability parameters. Besides all of this, the time needed for optimization, the expensive tools required, and the hazardous organic reagents utilized are considered potential obstacles to that technique^31^. On the other hand, spectrophotometric techniques can overcome these previously mentioned drawbacks as it is fast, cheap, simple and time saving^32^. To this end, our aim is to simultaneously analyze NZ, PN, and three official impurities, namely; NZ impurity B, PN impurity A and PN impurity B, using simple chemometrics-assisted spectrophotometric methods. Calibration mixtures are prepared based on five-level five-factor design. Partial least squares (PLS) and Artificial neural network (ANN) regressions are the chosen models for the determination of the five cited compounds (Fig. 1) in marketed dosage form and laboratory prepared mixtures.

## Results and discussion

**Figure 2:**
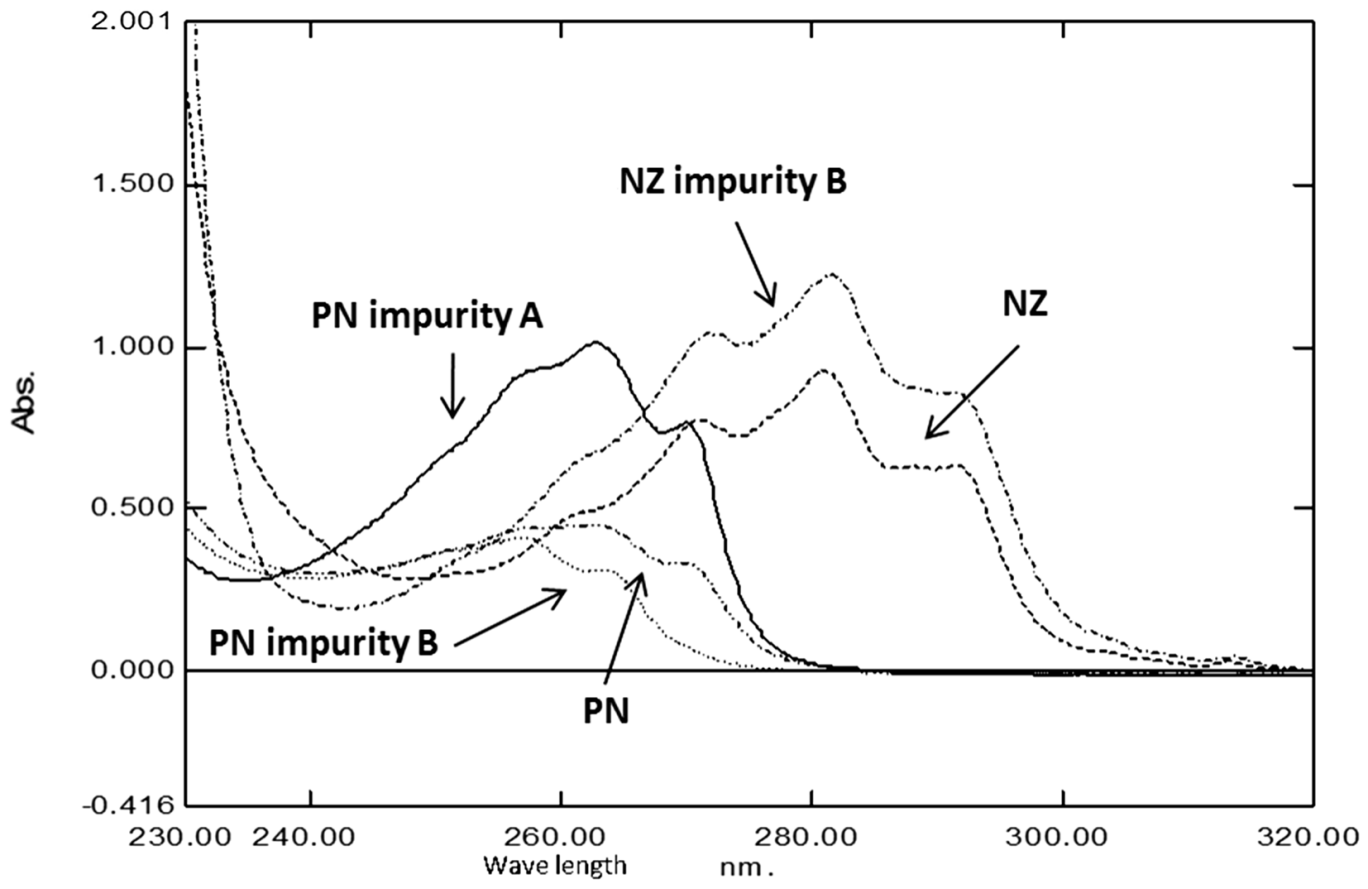
Normalized spectra of NZ (----), PN (${\_}..{\_}$), NZ impurity B (${\_}.{\_}$), PN impurity A (——) and PN impurity B (……) using methanol as solvent.

Among different analytical techniques used for drugs determination in their pharmaceutical formulations, spectrophotometry is the widely applicable one. This is attributed to simplicity and rapidity of spectro-analytical methods which has no need for neither sophisticated apparatus nor chemical pretreatment as other chromatographic methods^9^. Due to presence of sever overlapping between the spectra of the five studied components, Fig. 2. Multivariate spectrophotometric are the technique of choice to resolve this overlap. It was found that readings below 250.0 nm showed high noise which may affect the obtained result, otherwise readings above 300.0 nm gave almost zero absorption leading to invaluable information, thus the range between 250.0 and 300.0 nm was the range of choice in calculation. Brereton five-level calibration design was followed in order to prepare different mixtures of the five cited drugs^33^. 25 mixtures were prepared and divided into two groups. 15 mixtures for the calibration group whereas the remaining 10 mixtures were utilized for the validation one.

### Partial least squares model

**Figure 3:**
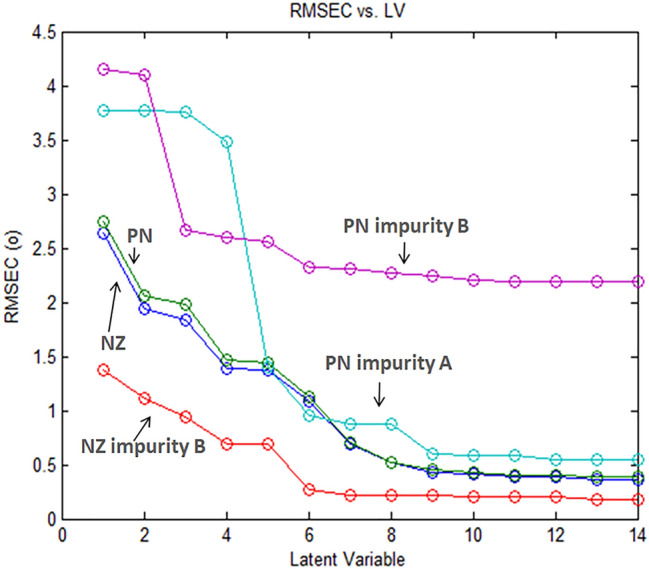
Root mean square error of calibration (RMSEC) versus the number of latent variables used to construct the PLS calibration for the assay of NZ, PN, NZ impurity B, PN impurity A and B in their mixtures.

During construction of PLS model, mean centering of all spectral data was adopted. After applying the leave-one-out as a cross-validation tool, latent variables’ number was obtained^6, 34^. The plot relating this number to root mean square error of calibration (RMSEC) revealed that six was the optimum number to be utilized, Fig. 3. It is worth noting that numerous models for calibration were tried and built whereas obtaining least noise as well as satisfactory recovery results were the parameters for selecting the optimum one^35^.

### Artificial neural network model

Three transferable layers (input, hidden and output) are usually incorporated in ANN model^8, 12^ In our model, the 251 points of spectral data were used as input neurons^11, 35^. On the other hand, 5 neurons related to number of drugs determined by such model comprised the output layer. For the hidden layer, various numbers were tried whereas RMSEC was calculated in each time. It was found that the lowest error (< 0.1 for the five drugs) was gained upon using 4 neurons in this layer with no further enhancement upon increasing their number. It is worth noting that pure-line transfer function, 0.1-learning rate as well as 50-epochs were also utilized.

The prediction’s capability of the two proposed models was verified by the aid of the validation group mixtures. For each drug, average recovery percentages as well as relative standard deviations were tabulated, Table 1. It is worth noting that the concentration ranges of the two drugs were chosen to facilitate their direct determination in the challenging eye drops ratio. Linearity parameters, limits of detection and quantification were obtained, Table 2. As shown, our proposed models could detect NZ and PN impurities at limits of about ≈1% and ≈2%, respectively, of their parent drugs' highest calibration concentration. Moreover and in order to test the presence of possible over-fitting, root mean square error of prediction (RMSEP) and standard error of prediction (SEP) were computed as well. As shown in Table 2, their close values assured over-fitting absence in our models.

**Table 1:** Prediction recoveries of the validation set samples by the constructed PLS and ANN models.

Mix no	NZ			PN			NZ impurity B			PN impurity A			PN impurity B		
	Actual conc µg mL^−1^	PLS	ANN	Actual conc µg mL^−1^	PLS	ANN	Actual conc µg mL^−1^	PLS	ANN	Actual conc µg mL^−1^	PLS	ANN	Actual conc µg mL^−1^	PLS	ANN
1	11.0	102.6	97.4	60.0	98.6	98.0	1.00	102.4	98.6	2.00	101.5	97.8	8.00	96.8	98.4
2	13.0	100.3	102.9	10.0	99.5	98.0	1.00	100.8	99.3	8.00	102.2	102.1	11.0	98.7	99.9
3	5.00	99.0	100.8	10.0	99.1	97.4	3.00	101.4	98.7	11.0	102.6	100.2	2.00	102.4	100.0
4	5.00	96.5	102.9	35.0	97.0	102.2	4.00	98.5	101.0	2.00	101.5	100.3	11.0	101.4	100.5
5	9.00	101.2	99.3	47.5	100.9	102.1	1.00	99.0	100.9	11.0	103.0	98.6	5.00	97.6	100.3
6	11.0	97.8	97.4	10.0	100.7	101.1	4.00	97.5	97.8	5.00	102.0	98.3	5.00	99.2	96.3
7	5.00	98.3	100.2	47.5	99.7	101.2	2.00	102.4	99.9	5.00	98.6	98.7	8.00	97.9	101.2
8	11.0	100.5	101.7	22.5	98.5	98.9	2.00	101.4	100.4	8.00	97.2	102.3	2.00	101.5	96.3
9	7.00	100.6	97.9	22.5	103.9	99.9	3.00	97.3	102.5	2.00	101.6	100.9	5.00	99.5	97.3
10	7.00	98.4	101.5	35.0	100.0	101.4	1.00	99.6	100.6	5.00	99.9	103.2	2.00	98.3	97.1
Mean ± SD		99.5 ± 1.8	100.2 ± 2.1	Mean ± SD	99.8 ± 1.8	100.0 ± 1.8	Mean ± SD	100.0 ± 1.9	100.0 ± 1.4	Mean ± SD	101.0 ± 1.9	100.2 ± 1.9	Mean ± SD	99.3 ± 1.9	98.7 ± 1.9

**Table 2:** Regression parameters of the validation sets calculated for each developed model.

Component	Model	Slope	Intercept	R	LOD (µg mL^−1^)	LOQ (µg mL^−1^)	SEP	RMSEP
NZ	PLS	1.0209	− 0.1881	0.9989	0.447	1.354	0.161549	0.144494
	ANN	1.0046	− 0.0224	0.9987	0.494	1.497	0.160227	0.143312
PN	PLS	0.9901	0.1796	0.9996	1.750	5.303	0.602116	0.538549
	ANN	1.0025	0.0281	0.9994	2.093	6.344	0.686162	0.613722
NZ impurity B	PLS	0.9739	0.0468	0.9994	0.138	0.419	0.056296	0.050353
	ANN	0.9959	0.0074	0.9994	0.144	0.435	0.046329	0.041438
PN impurity A	PLS	1.0245	− 0.0763	0.9992	0.454	1.375	0.190702	0.170569
	ANN	1.0010	0.0144	0.9994	0.384	1.163	0.12557	0.112314
PN impurity B	PLS	0.9925	− 0.0112	0.9994	0.389	1.180	0.14163	0.126678
	ANN	1.0084	− 0.0925	0.9997	0.277	0.839	0.106399	0.095166

### Eye drops application

Successful determination of NZ and PN in naphcon-A drops was attained by the proposed multivariate chemometrics models. As shown in Table 3, good recoveries were obtained and the models’ validity was further assured through recoveries of the added standards.

**Table 3:** Determination of NZ, PN in their dosage form and application of standard addition technique using the proposed PLS and ANN models.

Naphcon-A^®^ eye drop	% Found Mean^a^ ± SD	Standard addition technique				
		Taken		Added	Recovery%	
PLS						
NZ	99.6 ± 0.7	4.00 µg mL^−1^		2.00 µg mL^−1^		102.0
				4.00 µg mL^−1^		100.1
				8.00 µg mL^−1^		99.6
Mean ± SD						100.6 ± 1.3
PN	100.0 ± 0.8	10.0 µg mL^−1^		5.00 µg mL^−1^		100.3
				10.0 µg mL^−1^		100.4
				20.0 µg mL^−1^		101.1
Mean ± SD						100.6 ± 0.4
ANN						
NZ	99.9 ± 1.8	4.00 µg mL^−1^		2.00 µg mL^−1^		100.3
				4.00 µg mL^−1^		99.9
				8.00 µg mL^−1^		99.0
Mean ± SD						99.7 ± 0.7
PN	98.9 ± 1.3	10.0 µg mL^−1^		5.00 µg mL^−1^		100.2
				10.0 µg mL^−1^		99.3
				20.0 µg mL^−1^		100.0
Mean ± SD						99.8 ± 0.5

^a^Average determinations of four eye drop dosage form.

### Statistical analysis

Student’s t-test as well as F- one were statistically applied to compare obtained results with that acquired by BP methods of NZ and PN analyses. Table 4 summarizes the outputs of this comparison along with average recoveries, standard deviations, variances, and number of observations utilized in those tests. It is worth noting that the lower t and F values obtained relative to theoretical ones assured the statistical absence of significant differences.

**Table 4:** Statistical comparison between the results obtained by the proposed PLS and ANN models and the official BP method of analysis of NZ, PN.

Parameter	PLS		ANN		Official BP method^a^	
	NZ	PN	NZ	PN	NZ	PN
Mean	99.5	99.8	100.2	100.0	99.6	99.7
SD	1.82	1.84	2.12	1.83	0.98	1.15
Variance	3.31	3.39	4.49	3.35	0.96	1.32
n	10	10	10	10	5	5
Student’s t-test	0.11 (2.16)^b^	0.08 (2.16)^b^	0.56 (2.16)^b^	0.35 (2.16)^b^	–	–
F-test	3.48 (5.60)^b^	2.55 (5.60)^b^	4.72 (5.60)^b^	2.52 (5.60)^b^	–	–

^a^NZ was analyzed using stationary phase octylsilyl silica gel and mobile phase of sodium octane sulphonate in a mixture of 5.0 mL of glacial acetic acid, 300.0 mL of acetonitrile and 700.0 mL of water, flow rate 1.0 mL min^−1^ and detection at 280.0 nm. PN was analyzed using stationary phase C18 with gradient mobile phase of sodium heptane sulphonate pH 2.5 and acetonitrile, flow rate 1.0 mL min^−1^ and detection at 264.0 nm.

^b^These values represent the corresponding tabulated values of t and F at *p* = 0.05.

### Comparison with reported chromatographic methods

Comparing our proposed models with the reported chromatographic methods for the determination of NZ, PN, and the three selected impurities was conducted. The solvents used, the linearity ranges as well as the obtained LOD values were the chosen items for this comparison, Table 5. The table proved the superiority of these proposed PLS and ANN spectrophotometric models in terms of the simplicity of solvents required. Lower LOD values were also obtained compared to the reported HPLC method.

**Table 5:** Overview on the reported chromatographic methods for determination of the five cited components.

Ref. no	Technique	Utilized solvents	Linearity range	LOD
^28^	HPLC	Acetonitrile Phosphate buffer pH 6.0	NZ: 5–45 µg mL^−1^ PN: 10–110 µg mL^−1^ NZ impurity B: 5–45 µg mL^−1^ PN impurity A: 10–70 µg mL^−1^ PN impurity B: 10–120 µg mL^−1^	NZ: 1.29 µg mL^−1^ PN: 3.10 µg mL^−1^ NZ impurity B: 1.43 µg mL^−1^ PN impurity A: 1.98 µg mL^−1^ PN impurity B: 3.00 µg mL^−1^
^30^	TLC	Methanol Ethyl acetate 33.0% ammonia	NZ: 2–50 µg band^−1^ PN: 10–110 µg band^−1^ NZ impurity B: 0.1–10 µg band^−1^ PN impurity A: 0.2–50 µg band^−1^ PN impurity B: 0.2–50 µg band^−1^	NZ: 0.60 µg band^−1^ PN: 2.38 µg band^−1^ NZ impurity B: 0.01 µg band^−1^ PN impurity A: 0.05 µg band^−1^ PN impurity B: 0.06 µg band^−1^
This work	UV spectrophotometry	Methanol	NZ: 5–13 µg mL^−1^ PN: 10–60 µg mL^−1^ NZ impurity B: 1–50 µg mL^−1^ PN impurity A: 2–14 µg mL^−1^ PN impurity B: 2–14 µg mL^−1^	NZ^a^: 0.45 µg mL^−1^ PN^a^: 1.75 µg mL^−1^ NZ impurity B^a^: 0.14 µg mL^−1^ PN impurity A^a^: 0.38 µg mL^−1^ PN impurity B^a^: 0.28 µg mL^−1^

^a^Lower value was selected between the two proposed PLS and ANN models.

## Conclusion

In this work NZ, PN along with three selected official impurities are determined in their mixtures and eye drops pharmaceutical dosage form using accurate and simple chemometrics assisted spectro-analytical models. The proposed models are considered time-saving and cost-effective comparing with the reported chromatographic methods. The two proposed models show the advantage of the low values for prediction error. PLS supposes that the error is scattered equally between the two matrices; concentration and spectral response. As a result, robust results were given by this model through removing the absorbance as well as concentration data noises simultaneously. On the other hand, the ANN model’s predicting ability was exploited for obtaining more precise results during studied drugs’ quantification.

## Methods

### Instrument

A dual-beam Shimadzu, UV 1601 spectrophotometer, Kyoto, Japan. Spectra Scanning was conducted at 200.0–400.0 nm range with 0.2 nm intervals using 1.00-cm quartz cuvettes. The utilized Matlab^®^ software (7.0.1) was integrated with a PLS Toolbox 2.1.

### Materials

#### Standards

The studied drugs were supplied by Eva-pharma Co. (Egypt). Purities were checked as per BP methods to be 100.12% for NZ, and 99.58% for PN^15^. The impurities were purchased from Alfa Aesar Co. (Germany). Potencies were certified to be 99.00%, 100.30% and 99.70% for NZ impurity B, PN impurity A and PN impurity B, respectively.

#### Pharmaceutical dosage form

Naphcon-A drops, Alcon lab. INC. B. N. H13949-0615, containing 0.25 mg NZ and 3.0 mg PN in one mL.

#### Reagents

Methanol of analytical-grade was used (Alpha, Egypt).

#### Solutions

Five standard solutions, of 1.0 mg mL^−1^ concentration, were prepared separately using methanol.

### Procedures

#### Construction of the calibration models

Spectral features of the five studied substances were determined through their normalized spectra using methanol as a blank. Twenty-five mixtures containing different concentrations of the five cited compounds were prepared following a five-levels five-factors experimental design to reach concentration ranges of 5.0–13.0 μg mL^−1^ for NZ, 10.0–60.0 μg mL^−1^ for PN, 1.0–5.0 μg mL^−1^ for NZ impurity B and 2.0–14.0 μg mL^−1^ for two PN impurities. Scanning of the prepared solutions were then conducted at 200.0–400.0 nm range. The range from 250.0 to 300.0 nm was used for further calculations. The data were analyzed using Matlab^®^, where multivariate calibration models were built by using fifteen mixtures from the previously prepared solutions.

#### Validation of the calibrated models

The remaining ten mixtures from the previously prepared solutions were used for the validation purpose. PLS and ANN obtained parameters were used for quantification of the five cited compounds.

#### Application to naphcon-A drops

A 1.0-mL solution was transferred from naphcon-A drops to a 50-mL flask, and 25.0 mL methanol was introduced. Sonication was then applied for 10.0 min. A final concentration of 5.0 µg mL^−1^ NZ and 60.0 µg mL^−1^ PN was obtained after completing the volume with methanol. The prepared solutions were scanned and concentrations were predicted by the developed models.

## Data Availability

The data that support the findings of this study are available from the corresponding author upon reasonable request.
